# Rapid and Sensitive Multiplex Assay for the Detection of *B. anthracis* Spores from Environmental Samples

**DOI:** 10.3390/pathogens9030164

**Published:** 2020-02-27

**Authors:** Efi Makdasi, Orly Laskar, Itai Glinert, Ron Alcalay, Adva Mechaly, Haim Levy

**Affiliations:** 1 The Department of Infectious Diseases, Israel Institute for Biological Research, Ness Ziona 74100, Israel; Efim@iibr.gov.il (E.M.); Orlyl@iibr.gov.il (O.L.); Itaig@iibr.gov.il (I.G.); Advam@iibr.gov.il (A.M.); 2 The Department of Biochemistry and Molecular Genetics, Israel Institute for Biological Research, Ness Ziona 74100, Israel; Rona@iibr.gov.il

**Keywords:** *Bacillus anthracis*, anthrax, environment samples, multiplex, protective antigen, lethal factor, edema factor

## Abstract

Prompt and accurate detection of *Bacillus anthracis* spores is crucial in the event of intentional spore dissemination in order to reduce the number of expected casualties. Specific identification of these spores from environmental samples is both challenging and time-consuming. This is due to the high homology with other Bacillus species as well as the complex composition of environmental samples, which further impedes assay sensitivity. Previously, we showed that a short incubation of *B.anthracis* spores in a defined growth medium results in rapid germination, bacterial growth, and secretion of toxins, including protective antigen. In this work, we tested whether coupling the incubation process to a newly developed immune-assay will enable the detection of secreted toxins as markers for the presence of spores in environmental samples. The new immune assay is a flow cytometry-based multiplex that simultaneously detects a protective antigen, lethal factor, and edema factor. Our combined assay detects 1 × 10^3^–1 × 10^4^/mL spores after a 2 h incubation followed by the ~80 min immune-multiplex detection. Extending the incubation step to 5 h increased assay sensitivity to 1 × 10^2^/mL spore. The protocol was validated in various environmental samples using attenuated or fully virulent *B. anthracis* spores. There was no substantial influence of contaminants derived from real environmental samples on the performance of the assay compared to clean samples, which allow the unequivocal detection of 3 × 10^3^/mL and 3 × 10^2^/mL spores following 2 and 5 hour’s incubation, respectively. Overall, we propose this method as a rapid, sensitive, and specific procedure for the identification of *B. anthracis* spores in environmental samples.

## 1. Introduction

*Bacillus anthracis (B. anthracis)*, a gram-positive member of the *Bacillus cereus (B. cereus)* group, is the causal agent of anthrax, a serious and often fatal disease of animals and humans [1,2]. This pathogen is classified as a tier 1 bio-threat agent due to its highly pathogenic nature [3]. Anthrax spores are extremely stable in the environment as well as easy to disperse, making these agents extremely suitable for use as a bio-weapon. In humans, three types of anthrax disease have been recorded according to the route of infection: Cutaneous inoculation via a cut or abrasion, gastrointestinal infection resulting from the consumption of contaminated meat, and the inhalational form of the disease. Inhalational anthrax is a rare disease naturally, historically associated with industrial exposure to spores [2,4]. This form is also the most likely outcome of a deliberate release of aerosolized spores [1,5]. 

The virulence of *B. anthracis* relies on the presence of two virulence plasmids: pXO2, encoding the capsule operon, and pXO1, encoding the tripartite toxin genes. These include the protective antigen (PA), lethal factor (LF), and edema factor (EF) proteins. PA is a pore-forming protein, necessary for the translocation of the catalytic LF (metalloprotease specific for MAP kinases) and EF (calmodulin-dependent adenylate cyclase) into target mammalian cells. PA is non-toxic by itself, while LF and EF are inactive outside the host cell and depend on PA activity to function [1]. 

In the event of *B. anthracis* spores being used as a bio-weapon (such as a terror attack), a method enabling direct detection of spores in environmental samples can prove invaluable for accurate detection and risk assessment. Detection of *B. anthracis* in clinical or environmental samples is usually performed by applying one or more of the following methodologies; classical microbiological methods are still used as initial indications for the presence of the bacterium. These methods include gram stain, hemolysis and motility tests, capsule production, and gamma phage susceptibility [6]. Nucleic acid-based techniques, from PCR to whole genome sequencing and transcriptomics [7,8,9] are also applied. Immunoassays are widely applied for the detection of the bacterium, usually by using antibodies specific to the PA or capsule [10]. In addition, specific enzyme activity tests, usually of the LF [6], have also been developed. Though these assays are considered highly sensitive in homogeneous samples, their specificity and sensitivity are significantly hampered in complex environmental samples such as soil samples [11]. This can be related to the existence of ~2 × 10^9^ background bacteria/g of soil, usually of closely related species, which are found in the top 1 m ground layer [12]. In addition, the chemical and physical composition of environmental samples is complex, unknown, and highly variable, possibly containing substances that may impede the assays usually applied for spore detection [10]. The most direct way to overcome these problems is to isolate *B. anthracis* by plating the sample on selective agar media. This solution is time consuming and usually results in enrichment of the desired bacteria rather than absolute purification. Alternatively, spores could be purified from the samples using affinity-based methods [13,14,15,16,17,18,19]. The detection of *B. anthracis* spores from environmental samples in various immune affinity-based assays has been shown to be effective for the detection of 1 × 10^5^–1 × 10^6^ spores, in assays taking several hours to complete [11]. PCR based assays using specific primers and probes for the virulence plasmids were able to identify 1 × 10^5^ spores/g soil using multiplex-PCR and 1 × 10^3^–1 × 10^4^ spores/g soil using real-time PCR with assays that are somewhat faster, taking a few hours to complete [7,8,20]. The drawback of both PCR and antibody-based tests is the lack of ability to determine the viability of the spores. Currently, rapid viability PCR (RV-PCR) that combines qPCR with bacterial growth is the only diagnostic assay validated by the Environmental Protection Agency (EPA) for the detection of live spores. This test detects approximately 10 live spores in the presence of 1 × 10^6^ autoclaved spores within 17 h [21,22].

In this study, we examine the possibility of detecting the spores directly from the environmental sample by a rapid homogenous growth based test. Previously, it was shown that PA could serve as a surrogate marker for the estimation of the concentration of *B. anthracis* bacteria in animal models [23,24]. Moreover, we demonstrated that a short incubation of *B. anthracis* spores in Dulbecco’s Modified Eagle’s medium (DMEM) growth media supplemented with 10% serum in the presence of CO_2_ results in the rapid production and secretion of virulence factors during spore germination and initial bacterial growth, mainly PA [25].

Herein, we have applied these findings in developing a multiplexed flow cytometry (FCM) based assay to simultaneously detect PA, LF, and EF as surrogates for the presence of *B. anthracis* spores in environmental samples. The new assay includes two steps: An initial toxin biosynthesis step of 2–5 h, followed by a ~80 min immune-multiplex detection step of the secreted virulence factors. We demonstrate that this 4–7 h test is specific, sensitive, and can indirectly detect viable *B. anthracis* spores with a significant dynamic range, in environmental samples. 

## 2. Results

The proposed *B. anthracis* spore detection method is a two-step test where the environmental sample is cultured followed by immune-based detection of the tripartite toxin components in the growth medium. In order to achieve this goal we optimized the growth conditions to maximize toxin secretion, and developed a specific, sensitive multiplex test for the detection of the secreted toxins.

### 2.1. Development of a Magnetic Bead Immunoassay Based Flow Cytometry Method for the Detection of Anthrax Virulence Factors

**Figure 1:**
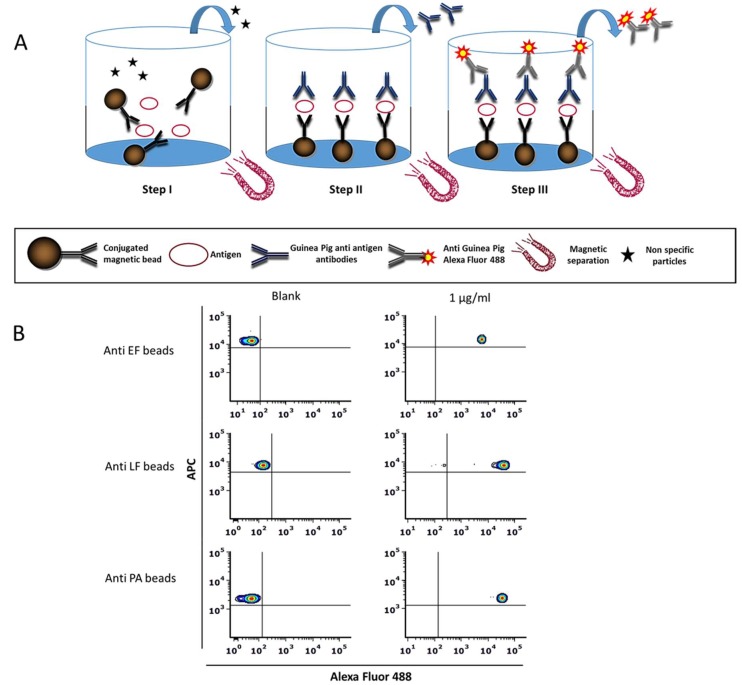
Magnetic bead immunoassay based flow cytometry method for the detection of protective antigen (PA), lethal factor (LF), and edema factor (EF). (**A**) Schematic representation of the assay developed in this study. The assay is comprised of three steps: Step I, incubation of the antigen-containing sample with a specific monoclonal antibody conjugated to magnetic beads. Step II, the addition of Guinea Pig anti-antigen antibodies, to form a sandwich immunoassay linked to the magnetic beads. Step III, the addition of anti-Guinea Pig IgG:A488 antibody. Each step is followed by a magnetic wash step in order to remove unbound components. Magnetically captured beads were resuspended in PBS + 1%BSA and analyzed by flow cytometry. (**B**) Specific color-code region 015/020/012 of magnetic beads characterized with unique fluorescence intensities (in the APC channel) were coupled separately with a specific antibody for EF (upper panels), LF (middle panels), and PA (lower panels), respectively, and were incubated in the presence of 1 µg/mL relevant antigen (right panels) or PBS + 1% BSA as blank (left panel). The upper left quadrant represents negative control, while the upper right quadrant represents positive staining.

The basic principle of the assay developed in this work is depicted in Figure 1A. In this study, three magnetic beads with different distinct fluorescence intensity profiles were used. These were coupled separately to specific monoclonal antibodies against EF, LF, or PA. In the presence of the relevant antigen, the Interaction of the antibody-coupled beads with the antigen results in a complex formation that can then be captured magnetically for washing out irrelevant proteins and particles throughout the assay. Consequently, labeling occurs by specific polyclonal IgG antibodies targeted against the antigen of interest (i.e., PA, LF, and EF). The resulting sandwich-complex is then detected through the addition of a secondary fluorescent-labeled antibody. FCM analysis allows the identification of the labeling-fluorophore in parallel to the beads’ fluorescence. Figure 1B shows a representative flow cytometry analysis of the three individual assays for the detection of EF, LF, and PA proteins separately.

**Figure 2:**
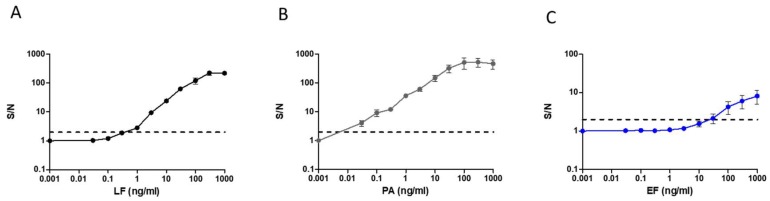
Standard curves of a magnetic bead-based immunoassay for the detection of (**A**) LF (**B**) PA and, (**C**) EF antigens. Purified antigens in different concentrations (0.03–1000 ng/mL) were analyzed separately in individual immunoassays. Results are presented as average ± SE (error bars) of signal to noise ratio (S/N) from three independent sets of measurement. Positive detection was determined as S/N ≥ 2 (black dashed line).

In order to characterize the assay sensitivity, dose-response assays were performed for the three individual antigens separately (Figure 2). The mean fluorescence intensity (MFI) for the resulting individual Alexa fluor 488 stained complexes was proportional to the concentration of the antigen in the sample. The results were calculated as the ratios between the signal (S) measured for each concentration compared to the signal measured with the assay run against antigen-free PBS (noise, N). This is termed the S/N ratio. S/N ration of 2 and above is considered a positive signal. Results demonstrate a wide dynamic range with a detection threshold of 0.03 ng/mL and 0.3–1 ng/mL for PA and LF, respectively. For EF, the assay proved less sensitive, and a positive signal was obtained from a concentration of 30–100 ng/mL.

The magnetic beads chosen for this assay are MegaPlex-C beads. These beads are unique as, in addition to being magnetic, they are available in a wide range of particular and distinct fluorescence intensities. These intensity level differences (hence “color-codes”) allow the separation of different bead sub-populations by FCM analysis using the 633 nm laser. This feature enabled the incorporation of the three individual assays into a single, multiplexed assay. Provided that sensitivity and specificity are not compromised, this format allows rapid and simultaneous detection of PA, LF, and EF virulence proteins in the same sample.

**Figure 3:**
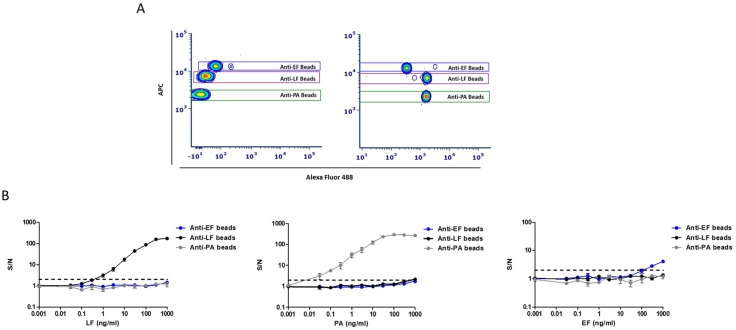
Standard curves of the multiplexed immunoassay for the detection of PA, LF, and EF antigens. (**A**) Dot plot analysis for the multiplexed assay in the negative (left) and positive (right) samples. Positive samples contain mixtures of PA (100 ng/mL), LF (100 ng/mL), and EF (1 µg/mL). Bead populations were separated according to their fluorescence intensity detected in the APC channel and gated (colored rectangles) for S/N calculation. (**B**) Purified antigens in different concentrations (0.03–1000 ng/mL) were analyzed separately in the multiplexed immunoassay. Results are presented as averages ± SE (error bars) of signal to noise ratio (S/N) from three independent sets of measurement. Positive detection was defined as S/N ≥ 2 (black dashed line).

To examine the FCM ability to distinguish between the different beads in a multiplex assay, we tested whether mixed beads can be separately gated by their color-code. Antigens (PA, LF, and EF) were added to one mixture (“positive control”) while the other was kept as a negative control (without antigens). Analysis of these samples showed that each individual bead population could be identified and separately gated (Figure 3A). Once we successfully demonstrated the ability to differentially identify discrete bead populations in multiplex format, we set out to estimate the effect of the multiplex format on the detection sensitivity of the assay. In order to assess assay sensitivity and specificity, multiplexed dose-response assays were performed with purified antigens. No significant changes in assay sensitivity (Figure 3B) for PA and LF were observed compared to the individual assays (Figure 2). A slight decrease in sensitivity was observed for EF detection. Importantly, no cross-reactivity was observed for LF and EF proteins. A minimal amount of cross-reactivity at high PA concentrations (1 µg/mL) with EF and LF coupled beads was measured. Since our purified PA antigen contained traces of bacterial proteins [26], we assumed that these impurities were the cause of the cross-reactivity. To ascertain assay specificity, a supernatant derived after a 24 h incubation of delta EF/LF Vollum strain [27] was tested (Supplementary Materials Figure S1). As hypothesized, no cross-reactivity of PA was observed with EF and LF coupled beads.

### 2.2. Applying the New Multiplex Assay for B. anthracis Spore Detection

**Figure 4:**
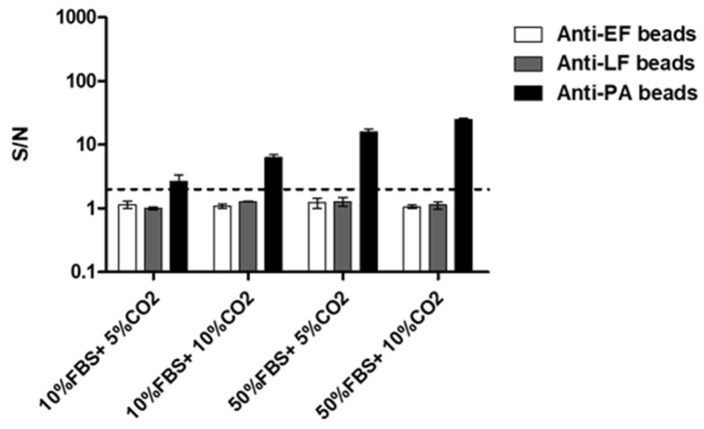
Optimal conditions for efficient production and secretion of toxins. *B. anthracis* ATCC14185 spores (5 × 10^5^ CFU/mL) were suspended in 400µl Dulbecco’s Modified Eagle’s medium (DMEM) supplemented with 10% or 50% fetal bovine serum (FBS) and incubated at 37 °C, under 5% or 10% CO_2_. Two hours later, the supernatants were analyzed for secreted EF, LF, and PA in the new multiplexed immune assay. Results are presented as averages ± SE (error bars) of signal to noise ratio (S/N) from two independent experiments. Positive detection was defined as S/N ≥ 2 (black dashed line).

Thus far, we demonstrated that our new multiplex immune-assay can simultaneously detect three secreted virulence factors of *B. anthracis*. This assay is rapid (approximately 80 min), sensitive, and specific. Previous research indicates that PA can serve as a surrogate marker to estimate the concentration of *B. anthracis* [23]. In addition, we have also demonstrated that a short incubation in DMEM (supplemented with 10% serum in the presence of CO_2_) results in germination and bacterial growth coupled with the early secretion of virulence factors [25,28]. Since this toxin production process is performed under batch conditions (microtiter dish), the concentration of these factors in the growth media increases over time. Therefore, potentially we can increase the toxin concentration in the growth medium by prolonging the incubation phase. Coupling this incubation process with sensitive and specific detection assays may enable spore detection in environmental samples containing low spore concentrations. Therefore, the proposed test combines sample incubation under increased toxin secretion conditions with the multiplex FCM based assay. In order to ascertain the optimal growth conditions enabling unequivocal identification of virulence factors in relation to the initial spore concentration, *B. anthracis* ATCC14185 spores (5 × 10^5^ CFU/mL) were suspended in medium containing 10% or 50% fetal bovine serum (FBS) and incubated at 37 °C for 2 h in the presence of 5% or 10% CO_2_. Following incubation, the presence of virulence factors in the growth medium was assessed by the multiplexed assay. Results (Figure 4) show detectable levels of PA following 2 h incubation while no significant detection of LF or EF was observed. Though positive detection of PA (S/N > 2) was obtained in all samples, the highest signal was reached in samples incubated in 50% FBS growth medium in 10% CO_2_. These were thus defined as the optimal conditions for increased toxins secretion. These results validate our hypothesis that PA is secreted in detectable quantities at the early stages of bacterial growth and is, therefore, suitable as a surrogate marker for spore detection.

**Figure 5:**
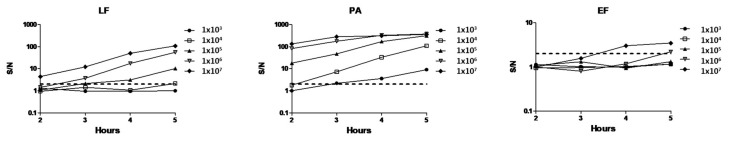
Extending the incubation process increases assay sensitivity. *B. anthracis* ATCC14185 spores in different initial concentrations (1 × 10^3^–1 × 10^7^ CFU/mL) were suspended in 400 µl DMEM supplemented with 50% FBS and incubated at 37 °C, under 10% CO_2_ for 2–5 h. Following incubation (toxin biosynthesis process), supernatants from each sample and time point were analyzed for the detection of secreted EF, LF, and PA in the new multiplexed immune assay. Results are presented as signal to noise ratios (S/N). Positive detection was defined as S/N ≥ 2 (black dashed line).

To evaluate the effect of extended incubation time on toxin secretion and assay sensitivity, different doses of ATCC14185 spores (1 × 10^3^–1 × 10^7^ CFU/mL) were incubated for 2–5 h under optimal conditions. Following the incubation process, toxin presence in the growth medium was determined by the multiplexed immunoassay. Our results (Figure 5) show that prolonged incubation time significantly improves the assay’s sensitivity. Similarly to the previous results, PA was the first to be detected in all samples. Positive detection of PA was possible within 2 and 3 h of incubation in 1 × 10^5^ and 1 × 10^3^–1 × 10^4^ spores/mL, respectively. Positive detection of LF was obtained after 3 h incubation for 1 × 10^5^ and 5 h for 1 × 10^4^ spores/mL. EF was detected only at high spore concentrations (above 1 × 10^6^ CFU/mL), after 4–5 h incubation. These results emphasize the benefit of using a dynamic process, in which samples can be collected continually along 5 h of incubation, thus increasing assay sensitivity. 

**Figure 6:**
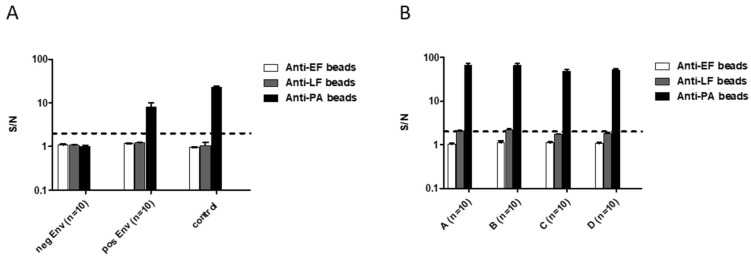
Identification of *B. anthracis* ATCC14185 spores in environmental samples. Environment samples collected from asphalt-paved roads, sidewalks, and various soils were spiked with 5 × 10^3^–1 × 10^4^ CFU/mL *B. anthracis* 14185 spores per sample. Samples were processed and resuspended in 400 µl DMEM supplemented with 50% FBS and incubated at 37 °C in 10% CO2 for 5 h. Following incubation (toxins biosynthesis process), supernatants from each sample were analyzed for secreted EF, LF, and PA in the new multiplexed immune assay. (**A**) Detection of EF/LF/PA was assessed following the incubation process of environmental samples (n = 10) spiked with 5 × 10^3^ CFU *B. anthracis* spores, negative environmental samples (n = 10), and 5 × 10^3^ CFU spores suspended in growth medium as a control. (**B**) Detection of EF/LF/PA was assessed, followed by an incubation process of 40 different environmental samples collected from four different geographic locations in Israel (marked as A-D) spiked with 1 × 10^4^ CFU *B. anthracis* spores. Results are presented as averages ± SE (error bars) of signal to noise ratios (S/N). Positive detection was defined as S/N ≥ 2 (black dashed line).

Since this method is supposed to detect spores in environmental samples, we tested our protocol on samples taken from asphalt-paved roads, sidewalks, and soil samples, which were spiked with known spore doses. First, we tested the effect of various environmental samples on the assay noise level in our multiplex assay. To this end, 10 environmental samples were incubated in a growth medium at 37 °C 10% CO_2_ for 5 h. Following incubation, the growth medium was tested for the presence of EF/LF/PA by the multiplex assay. No positive result was obtained in any of the negative samples (Figure 6A). Next, we assessed the effect of environmental contaminants on *B. anthracis* toxins secretion during the incubation process. Environmental samples (n = 10) were spiked with 5 × 10^3^ CFU/mL *B. anthracis* ATCC14185 spores, processed (Material and Methods), resuspended in growth medium and incubated at 37 °C 10% CO_2_ for 5 h. All environmental samples (Figure 6) were positive for PA (S/N range 4–25). However, the spiked environmental samples reached a lower signal (~2 fold decrease) relative to the same spore concentration spiked into PBS. This reduction may result from decreased spore dose in the final sample due to the spiking/processing protocol used, by signal inhibition resulting from the environmental sample constituents, or a combination of both. To validate these results, 40 additional environmental samples, collected in four separate locations in Israel, were spiked with 1 × 10^4^ CFU/mL spores and processed. Following 5 h incubation, PA was detected in all samples, while LF was detected only in 15/40 samples (S/N range 2–3, Figure 6B). In agreement with the above, PA levels significantly varied between samples, but all were clearly positive (S/N range 18–102, Figure 6B), indicating that the method is indeed suitable for *B. anthracis* spore detection in an environmental sample.

### 2.3. Assay Validation Using Fully Virulent B. anthracis Vollum Strain Spores

**Figure 7:**
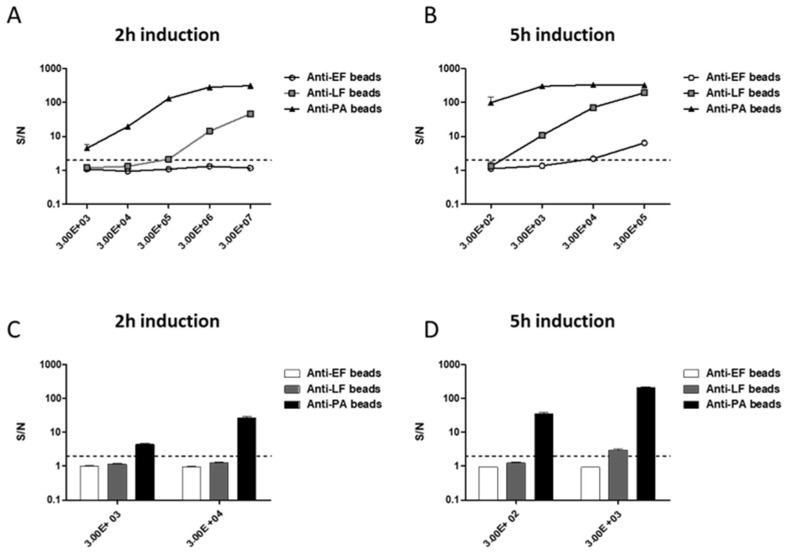
Toxins production and detection of virulent Vollum spores in the soil sample. Vollum spores in different concentrations (1 × 10^2^–1 × 10^7^ CFU/mL) were suspended in 400µl DMEM supplemented with 50% FBS and incubated at 37 °C, under 10% CO_2_ for (**A**) 2 h and (**B**) 5 h. Soil samples were spiked with 1 × 10^2^–1 × 10^4^ CFU spores/gram. Samples were processed and resuspended in growth medium for (**C**) 2 h and (**D**) 5 h at 37 °C under 10% CO_2_. Following incubation, supernatants from each sample in each time point were analyzed for the detection of EF, LF, and PA in the multiplexed immune assay. Results are presented as averages ± SE (error bars) of signal to noise ratios (S/N) from two independent experiments for A-B and 14 different soil samples for C-D. Positive detection was defined as S/N ≥ 2 (black dashed line).

Having performed all of our experiments on *B. anthracis* ATCC14185 spores, we validated our results using a fully virulent Vollum strain. Vollum spores (1 × 10^2^–1 × 10^7^ CFU/mL) were suspended in growth medium and incubated for 2 and 5 h. Results in Figure 7A show positive detection of PA and LF at concentrations of 1 × 10^3^ CFU/mL and 1 × 10^5^ CFU/mL spores, respectively, following a 2 h incubation process. Prolonging incubation process to 5 h enables the detection of PA at concentrations of 1 × 10^2^ CFU/mL and LF at concentrations of 1 × 10^3^ CFU/mL. A positive signal for EF was observed only from 1 × 10^4^ CFU/mL (Figure 7B). Positive PA detection was achieved in 14/14 different soil samples spiked with 1 × 10^3^ CFU Vollum spores/g after 2 h incubation (S/N range 3.5–5.5, Figure 7C). Prolonging incubation to 5 h (Figure 7D) resulted in detection of PA in soil samples spiked with 1 × 10^2^ CFU spores (S/N range 17–35). LF was observed (S/N range 2.5–4) in all 1 × 10^3^ CFU spores samples following 5 h. The assay proved more sensitive to the presence of Vollum spores compared to ATCC14185 spores. This could result from differences in toxin secretion between the two strains. The ATCC14185 strain is a vaccine strain, used to produce PA-containing drug substance and was mutated to facilitate this application [29]. These mutations may explain this difference.

Overall, the proposed method combining biosynthesis and identification of toxins can serve as a rapid and sensitive assay for the indirect detection of viable *B. anthracis* spores in environmental samples.

## 3. Discussion

*B. anthracis*, the causative agent of anthrax, has very close relatives in the *B. cereus* group [30], with which it shares a great deal of morphological, biochemical, and genetic similarities [31]. The background presence of these soil bacteria complicates the specific identification of *B. anthracis* in environmental samples. Among the few features distinguishing *B. anthracis* within the *B. cereus* group (excluding biovar anthracis [32]), is the presence of two virulence plasmids: pXO1, encoding the tripartite toxin genes and pXO2, encoding the capsule operon. The tripartite toxin is comprised of PA, LF, and EF [2]. 

*B. anthracis* is one of the most notorious infectious agents, and thus remains classified as a tier 1 bio-threat affecting both livestock and humans [3]. Its ability to produces highly resistant spores, the fact that it is endemic (and, therefore, available for isolation and culture) in most of the world, and that the spores are easily dispersed, all make this bacterium highly suitable for weaponization [2].

In the scenario of a deliberate release of anthrax spores, diagnostic efforts will focus on two types of samples—clinical samples from suspected patients and environmental samples from suspected contaminated areas. In this work, we focused on the development of a method that enables sensitive detection of spores in environmental samples. Such an assay will be useful both in initial detection and risk assessment as well in subsequent decontamination efforts.

We demonstrated that the optimal conditions for the incubation process were determined to be in supplemented DMEM containing 50% serum (hence growth medium) under 10% CO_2_. Furthermore, this incubation protocol, combined with the immunoassay, enables the detection of 1 × 10^3^–1 × 10^4^/mL spore concentration following a short, 2-hour incubation. Extending the incubation step to 5 hours increased assay sensitivity to allow the detection of 1 × 10^2^/mL spore. The extended protocol was validated in 50 various environmental samples spiked in 5 × 10^3^–1 × 10^4^ CFU/mL *B. anthracis* 14,185 spores. All spiked samples were found unequivocally positive. In addition, 2 h incubation of 14 soil samples spiked with the fully virulent *B. anthracis* BA3500 spores resulted in the detection of 3 × 10^3^/mL spores. Extending the incubation to 5 h resulted in the detection of 3 × 10^2^/mL spores. Our assay has not been tested for more prolonged incubations, but it stands to reason that prolonging incubation duration will improve sensitivity. 

Dealing with environmental samples has proven to be challenging. Existing assays have been shown to suffer from limited assay specificity [33] and/or sensitivity [34]. Assays usually include specific selection and isolation of *B. anthracis* colonies, a process that may take days. Additional identification assays have low sensitivities for environmental samples, suffering a 3–4 orders of magnitude decrease in sensitivity compared to pure samples [10]. This was demonstrated using specific phage [35] or other commercial assays [36]. The detection of *B. anthracis* spores from environmental samples in various immune affinity-based assays has been shown to be effective for the detection of 1 × 10^5^–1 × 10^6^ spores [11,36]. In addition, using specific reporter phage enables the detection of 1 × 10^5^ CFU in assays, taking several hours to completion [35]. Regarding the specificity of these assay, caution is required, as antibodies against *B. anthracis* can also cross-react with other *B. cereus*-group strains [37]. PCR-based assays allow for the identification of ~1 × 10^5^ spores/g soil using multiplex-PCR and 1 × 10^3^–1 × 10^4^ spores/g soil using real-time PCR [7,8,20]. A highly sensitive PCR-based assay, detecting ~10 live spores in the presence of 1 × 10^6^ autoclaved spores, requires ~17 h to perform [21,22]. Our results demonstrate that in this assay, the presence of an environmental contaminant did not prove detrimental to the germination process, bacterial growth, virulence factor secretion, or the ability to achieve positive detection.

The newly assay is comprised of two steps: The incubation period, as described above, and the newly multiplexed magnetic beads immunoassay. The latter is comprised of three individual incubation steps for antigen capture and the labeling prior to FCM analysis, totaling an 80 min assay. The multiplexed nature of the assay allows for simultaneous detection of PA, LF, and EF in a small sample volume, which allows dynamic and continuous measurement of the secreted virulent factors during the incubation process. This assay applies Luminex particles. The application of such particle for FCM was previously described by Vignali [38] as a sensitive, simple, and reliable multiplexing method for the quantitation of cytokine, while specific use of Luminex particles for the detection of *B. anthracis* was previously described by Mechaly et al. [39]. This assay described the identification of soluble capsule and PA as specific markers in homogenous blood culture using the xMAP technology by Luminex.

In contrast to the classical microbiological methods, the short incubation process in our new assay enables the secretion of the *B. anthracis* virulent factors without a substantial influence on the biological and chemical composition of the environmental sample. Another positive effect of the incubation step is the proliferation of the bacteria. This proliferation should prove beneficial to additional diagnostic measures, such as classical immunological assays (i.e., immuno-fluorescent assay) and nucleic acid-based detection. 

We, therefore, propose that the newly developed methodology described in this work allows the detection of spore concentrations that are 3–4 orders of magnitude lower than published immuno-affinity assays [11,36], being comparable to sensitive PCR-based assays [7,8,20], while being performed in under 3.5 h for the total assay. This time frame could potentially be shortened even further by modifying the multiplex assay step to be performed as a one-step assay (all in one) by using direct labeled secondary antibodies or a biotin-streptavidin complex for signal enhancement. We plan to pursue this possibility in the near future. Such a sensitive, specific, and relatively rapid test may prove invaluable not only in the identification of the initial release of the threat agent, but also in the extensive forensic investigation following such an event and even play a part in monitoring the effectiveness of decontamination efforts.

Outside the realm of bio-terrorism, this assay may prove valuable in epidemiological studies following natural outbreaks involving livestock and pastures, allowing the analysis of soil samples pertaining to such events.

## 4. Materials and Methods

### 4.1. B. anthracis Strains

The strains used in this study were *B. anthracis* Vollum (ATCC 14578) (pXO1^+^ pXO2^+^) and Vollum Δ*lef*Δ*cya* [27]. ATCC 14185 (pXO1^+^ pXO2^-^), which is a non-encapsulated attenuated strain that produces the pXO1 encoded virulence factors, including PA, LE, and EF, was also used. All strains were from the Israel Institute for Biological Research collection.

### 4.2. Antibodies and Proteins 

The monoclonal antibodies utilized in this study were mouse anti-PA (m55) [40], mouse anti-EF (EMN2/1), and mouse anti-LF (LMN 1/5) [27]. These antibodies were purified from a mouse ascetic fluid using protein G chromatography (GE Healthcare, Uppsala, Sweden) according to the manufacturer’s instructions and dialyzed against phosphate-buffered saline (PBS; PH7.4; Biological Industries, Beth Haemek, Israel). Anti EF, LF, and PA polyclonal antibodies were obtained from hyperimmune guinea pigs immunized with purified EF, LF, or PA, respectively. Polyclonal antibodies were used as a serum. Donkey anti guinea pig IgG (H+L) conjugated to Alexa fluor 488 was purchased from Jackson immune research laboratories (cat #706-545-148). PA was purified, as described previously [41]. EF and LF were isolated by genetically inserting a His-tag sequence at the carboxy terminus of each protein in the background of the ATCC 14185 strain. Separate strains were produced for the purification of EF and LF. The proteins were purified on a nickel-column from the supernatant of bacterial cultures, as described in Reuveny et al. 2001 [41]. 

### 4.3. Preparation of Coupled Magnetic Beads 

In this study, we used Luminex MagPlex-C microspheres, 2.5 × 10^6^/mL, regions 12, 15, and 20 (Luminex, Austin, TX; catalog numbers MC10012, MC10015 and MC10020). Specific antibodies were coupled to beads (5 µg of each per 1 × 10^6^ beads) using the Luminex antibody coupling kit (catalog number 40-50016) according to the manufacturer’s instruction. 

### 4.4. Magnetic Bead Based Detection Assays

The optimization of the magnetic bead-based assay for each of the individual antigens tested was done separately. Assays were performed in a final volume of 100 μl in 96-well Greiner black microplates (Greiner, PP F-Bottom chimney well, 655209). The assays were comprised of three steps; 1. Coupled beads (5 × 10^4^) were mixed with 100 μl of either purified antigen or samples following the incubation process. The mixtures were then agitated in the dark at room temperature for 30 min in an orbital micro shaker (Dynatech, England). The plates were then washed by a microplate washer (Tecan, Hydrospeed, 30054550) equipped with a smart-2MBS 96 well magnetic plate. 2. Captured beads were re-suspended in 100 μl of guinea pig polyclonal IgG antibodies (diluted 1:1000 in PBS 1% BSA 0.05% tween) targeted against the specific antigen of interest and incubated for 30 min. 3. Following an additional magnetic wash, anti-Guinea Pig IgG:A488 (1:800) was added to the captured beads for an additional 10 min incubation. Beads were magnetically washed and resuspended in 200 μl PBS 1% BSA for FCM analysis using a BDTM Fortesa flow cytometer. Multiplexed assays were carried out as described for individual assays using 2 prepared assay mixtures containing the antibody-coupled beads and the anti-antigen polyclonal antibodies.

### 4.5. Environmental Sample Collection, Preparation, and Toxin Biosynthesis Process

Environmental samples were collected in 4 distinct locations. Samples included asphalt paved roads, sidewalks, and soil samples. As for the asphalt paved roads and sidewalks, samples were collected from 20 cm^2^ areas using two swabs soaked in 1× PBS, followed by one dry swab. At this stage, the swabs were inoculated with *B. anthracis* spores. The swabs were then vortexed in 4 mL PBS for 1 min, and 1 mL from the suspension was transferred to a clean 1.5 mL tube. Soil samples were comprised of 1 gram soil per sample. Samples were suspended in 1 mL PBS containing *B. anthracis* spores. All samples were vortexed, allowed 5 min incubation at room temperature (RT), prior to supernatants being transferred to new tubes, and centrifuged at 14,000 g for 5 min. Supernatants were discarded, and samples were resuspended in 400 µl growth medium containing DMEM + 50% Fetal Bovine Serum (FBS). The suspended samples were then plated on 48 well plates for 2–5 h incubation at 37 °C under 10% CO_2_.

### 4.6. Signal Analysis

The mean fluorescence intensity (MFI) for the resulting individual Alexa fluor 488 stained complexes was proportional to the concentration of the relevant antigen in the sample. The results were calculated as the ratios between the signal (S) measured for each concentration compared to the signal measured with the assay run against antigen-free PBS (noise, N). This was termed the S/N ratio. This calculation enabled the normalization of multiple experiments and the determination of a universal threshold for positive samples, in accordance with the International Council for Harmonization of Technical Requirements for Pharmaceuticals for Human Use (ICH) guidelines for validation of analytical procedures [42].

## Supplementary Materials

The following are available online at https://www.mdpi.com/2076-0817/9/3/164/s1, Figure S1: Specific detection of PA in a multiplexed immune assay.

## References

[1] Dixon T.C., Meselson M., Guillemin J., Hanna P.C. (1999). Anthrax. N. Engl. J. Med..

[2] WHO (2008). Anthrax in Humans and Animals.

[3] Tier1. www.selectagents.gov/ohp-app1.html.

[4] Brachman P.C. (1980). Inhalation Anthrax. Ann. N. Y. Acad. Sci..

[5] Jernigan D.B., Raghunathan P.L., Bell B.P., Brechner R., Bresnitz E.A., Butler J.C., Cetron M., Cohen M., Doyle T., Fischer M. (2002). Investigation of bioterrorism-related anthrax, United States, 2001: Epidemiologic findings. Emerg. Infect. Dis..

[6] LRN https://www.asm.org/Articles/Policiy/Laboratory-Response-Network-LRN-Sentinel-Level-C.

[7] Israeli O., Cohen-Gihon I., Zvi A., Lazar S., Shifman O., Levy H., Tidhar A., Beth-Din A. (2019). Rapid identification of unknown pathogens in environmental samples using a high-throughput sequencing-based approach. Heliyon.

[8] Ryu C., Lee K., Yoo C., Seong W.K., Oh H.B. (2003). Sensitive and rapid quantitative detection of anthrax spores isolated from soil samples by real-time PCR. Microbiol. Immunol..

[9] Be N.A., Thissen J.B., Gardner S.N., McLoughlin K.S., Fofanov V.Y., Koshinsky H., Ellingson S.R., Brettin T.S., Jackson P.J., Jaing C.J. (2013). Detection of *Bacillus anthracis* DNA in complex soil and air samples using next-generation sequencing. PLoS ONE.

[10] Irenge L.M., Gala J.L. (2012). Rapid detection methods for *Bacillus anthracis* in environmental samples: A review. Appl. Microbiol. Biotechnol..

[11] King D., Luna V., Cannons A., Cattani J., Amuso P. (2003). Performance assessment of three commercial assays for direct detection of *Bacillus anthracis* spores. J. Clin. Microbiol..

[12] Whitman W.B., Coleman D.C., Wiebe W.J. (1998). Prokaryotes: The unseen majority. Proc. Natl. Acad. Sci. USA.

[13] Dang J.L., Heroux K., Kearney J., Arasteh A., Gostomski M., Emanuel P.A. (2001). Bacillus spore inactivation methods affect detection assays. Appl. Environ. Microbiol..

[14] Zahavy E., Fisher M., Bromberg A., Olshevsky U. (2003). Detection of frequency resonance energy transfer pair on double-labeled microsphere and *Bacillus anthracis* spores by flow cytometry. Appl. Environ. Microbiol..

[15] Hang J., Sundaram A.K., Zhu P., Shelton D.R., Karns J.S., Martin P.A., Li S., Amstutz P., Tang C.M. (2008). Development of a rapid and sensitive immunoassay for detection and subsequent recovery of *Bacillus anthracis* spores in environmental samples. J. Microbiol. Methods.

[16] Tamborrini M., Holzer M., Seeberger P.H., Schurch N., Pluschke G. (2010). Anthrax spore detection by a luminex assay based on monoclonal antibodies that recognize anthrose-containing oligosaccharides. Clin. Vaccine Immunol..

[17] Yu H. (1998). Comparative studies of magnetic particle-based solid phase fluorogenic and electrochemiluminescent immunoassay. J. Immunol. Methods.

[18] Kuehn A., Kovac P., Saksena R., Bannert N., Klee S.R., Ranisch H., Grunow R. (2009). Development of antibodies against anthrose tetrasaccharide for specific detection of *Bacillus anthracis* spores. Clin. Vaccine Immunol..

[19] Sastry K.S., Tuteja U., Santhosh P.K., Lalitha M.K., Batra H.V. (2003). Identification of *Bacillus anthracis* by a simple protective antigen-specific mAb dot-ELISA. J. Med. Microbiol..

[20] Cheun H.I., Makino S.I., Watarai M., Erdenebaatar J., Kawamoto K., Uchida I. (2003). Rapid and effective detection of anthrax spores in soil by PCR. J. Appl. Microbiol..

[21] Letant S.E., Kane S.R., Murphy G.A., Alfaro T.M., Hodges L.R., Rose L.J., Raber E. (2010). Most-probable-number rapid viability PCR method to detect viable spores of *Bacillus anthracis* in swab samples. J. Microbiol. Methods.

[22] Kane S.R., Letant S.E., Murphy G.A., Alfaro T.M., Krauter P.W., Mahnke R., Legler T.C., Raber E. (2009). Rapid, high-throughput, culture-based PCR methods to analyze samples for viable spores of *Bacillus anthracis* and its surrogates. J. Microbiol. Methods.

[23] Kobiler D., Weiss S., Levy H., Fisher M., Mechaly A., Pass A., Altboum Z. (2006). Protective antigen as a correlative marker for anthrax in animal models. Infect. Immun..

[24] Cote C.K., Rossi C.A., Kang A.S., Morrow P.R., Lee J.S., Welkos S.L. (2005). The detection of protective antigen (PA) associated with spores of *Bacillus anthracis* and the effects of anti-PA antibodies on spore germination and macrophage interactions. Microb. Pathog..

[25] Levy H., Glinert I., Weiss S., Sittner A., Schlomovitz J., Altboum Z., Kobiler D. (2014). Toxin-independent virulence of *Bacillus anthracis* in rabbits. PLoS ONE.

[26] Glinert I., Bar-David E., Sittner A., Weiss S., Schlomovitz J., Ben-Shmuel A., Mechaly A., Altboum Z., Kobiler D., Levy H. (2016). Revisiting the Concept of Targeting Only *Bacillus anthracis* Toxins as a Treatment for Anthrax. Antimicrob. Agents Chemother..

[27] Levy H., Weiss S., Altboum Z., Schlomovitz J., Glinert I., Sittner A., Shafferman A., Kobiler D. (2012). Differential contribution of *Bacillus anthracis* toxins to pathogenicity in two animal models. Infect. Immun..

[28] Levy H., Glinert I., Weiss S., Bar-David E., Sittner A., Schlomovitz J., Altboum Z., Kobiler D. (2014). The central nervous system as target of *Bacillus anthracis* toxin independent virulence in rabbits and guinea pigs. PLoS ONE.

[29] Cohen-Gihon I., Israeli O., Beth-Din A., Levy H., Cohen O., Shafferman A., Zvi A., Chitlaru T. (2014). Whole-Genome Sequencing of the Nonproteolytic *Bacillus anthracis* V770-NP1-R Strain Reveals Multiple Mutations in Peptidase Loci. Genome. Announc..

[30] Helgason E., Okstad O.A., Caugant D.A., Johansen H.A., Fouet A., Mock M., Hegna I., Kolsto A.B. (2000). *Bacillus anthracis, Bacillus cereus*, and *Bacillus thuringiensis*—One species on the basis of genetic evidence. Appl. Environ. Microbiol..

[31] Ash C., Farrow J.A., Dorsch M., Stackebrandt E., Collins M.D. (1991). Comparative analysis of *Bacillus anthracis*, *Bacillus cereus*, and related species on the basis of reverse transcriptase sequencing of 16S rRNA. Int. J. Syst. Bacteriol..

[32] Brezillon C., Haustant M., Dupke S., Corre J.P., Lander A., Franz T., Monot M., Couture-Tosi E., Jouvion G., Leendertz F.H. (2015). Capsules, toxins and AtxA as virulence factors of emerging *Bacillus cereus* biovar anthracis. PLoS Negl. Trop. Dis..

[33] Tengelsen L., Hudson R., Barnes S., Hahn C. (2002). Coordinated response to reports of possible anthrax contamination, Idaho, 2001. Emerg. Infect. Dis..

[34] Lim D.V., Simpson J.M., Kearns E.A., Kramer M.F. (2005). Current and developing technologies for monitoring agents of bioterrorism and biowarfare. Clin. Microbiol. Rev..

[35] Sharp N.J., Molineux I.J., Page M.A., Schofield D.A. (2016). Rapid Detection of Viable *Bacillus anthracis* Spores in Environmental Samples by Using Engineered Reporter Phages. Appl. Environ. Microbiol..

[36] Ramage J.G., Prentice K.W., DePalma L., Venkateswaran K.S., Chivukula S., Chapman C., Bell M., Datta S., Singh A., Hoffmaster A. (2016). Comprehensive Laboratory Evaluation of a Highly Specific Lateral Flow Assay for the Presumptive Identification of *Bacillus anthracis* Spores in Suspicious White Powders and Environmental Samples. Health Secur..

[37] Tamborrini M., Oberli M.A., Werz D.B., Schurch N., Frey J., Seeberger P.H., Pluschke G. (2009). Immuno-detection of anthrose containing tetrasaccharide in the exosporium of *Bacillus anthracis* and *Bacillus cereus* strains. J. Appl. Microbiol..

[38] Vignali D.A. (2000). Multiplexed particle-based flow cytometric assays. J. Immunol. Methods.

[39] Mechaly A., Vitner E., Levy H., Weiss S., Bar-David E., Gur D., Koren M., Cohen H., Cohen O., Mamroud E. (2018). Simultaneous Immunodetection of Anthrax, Plague, and Tularemia from Blood Cultures by Use of Multiplexed Suspension Arrays. J. Clin. Microbiol..

[40] Rosenfeld R., Marcus H., Ben-Arie E., Lachmi B.E., Mechaly A., Reuveny S., Gat O., Mazor O., Ordentlich A. (2009). Isolation and chimerization of a highly neutralizing antibody conferring passive protection against lethal *Bacillus anthracis* infection. PLoS ONE.

[41] Reuveny S., White M.D., Adar Y.Y., Kafri Y., Altboum Z., Gozes Y., Kobiler D., Shafferman A., Velan B. (2001). Search for correlates of protective immunity conferred by anthrax vaccine. Infect. Immun..

[42] ICH (1996). ICH Harmonised Tripartite Guideline. Validation of Analytical Procedures: Text and Methodology.

