# Supplementary material for: Rapid and Sensitive Multiplex Assay for the Detection of B. anthracis Spores from Environmental Samples
Source: Pathogens. 2020 Feb 27;9(3):164. doi: 10.3390/pathogens9030164 (PMC7157734; doi:10.3390/pathogens9030164)
Supplement: Supplementary file 1 [file pathogens-09-00164-s001.pdf]

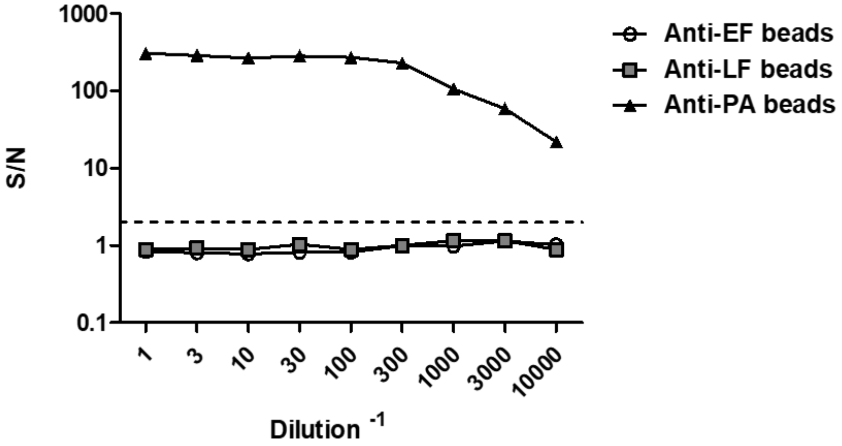

Supplemental figure 1. Specific detection of PA in a multiplexed immune assay. A ΔEF/LF Vollum strain (19) was incubated in induction growth medium at 37°C, 10% CO_2. 24 hours later supernatant was separated and diluted 1:3-1:10000 in PBS+1%BSA and samples were analyzed for the detection of secreted EF, LF and PA in the developed multiplexed immune assay. Results are presented as signal to noise ratios (S/N) from a representative experiment in two independent sets of measurement. Positive detection was defined as S/N≥2 (black dashed line).
